# Health simulation through the lens of self-determination theory — opportunities and pathways for discovery

**DOI:** 10.1186/s41077-024-00304-4

**Published:** 2024-07-22

**Authors:** Ellen Davies

**Affiliations:** Adelaide Health Simulation, Faculty of Health and Medical Sciences, The University of Adelaide, Adelaide, Australia

**Keywords:** Self-determination theory, Health simulation, Theory, Simulation-based education

## Abstract

Health simulation is broadly viewed as an appealing, impactful, and innovative enhancement for the education and assessment of health professions students and practitioners. We have seen exponential and global growth in programmes implementing simulation techniques and technologies. Alongside this enthusiasm and growth, the theoretical underpinnings that might guide the efficacy of the field have not always been considered. Many of the principles that guide simulation design, development and practice have been intuited through practical trial and error. In considering how to retrofit theory to practice, we have at our disposal existing theories that may assist with building our practice, expertise, identity as a community of practice, authority and legitimacy as a field. Self-determination theory (SDT) is an established and evolving theory that examines the quality of motivation and human behaviours. It has been applied to a variety of contexts and provides evidence that may support and enhance the practice of health simulation. In this paper, SDT is outlined, and avenues for examining the fit of theory to practice are suggested. Promising links exist between SDT and health simulation. Opportunities and new pathways of discovery await.

“A theory is a generative framework that not only enhances our understanding of phenomena, but also yields predictive principles that can anticipate solutions to new problems and novel applications” [1]

## Introduction

“Ok everyone, we’re here to do a sim, this is a safe space, nothing that is said or done here will leave the room”. “You’ve all done the work, you’ll all be fine. Just go in there and I’m sure you’ll be fabulous”. “This is a safe place to make mistakes—no patients will be harmed”. There is little doubt that people who have navigated themselves to this paper will have either said or heard these words in the context of health simulation. These words are quite comforting to say, and we genuinely want them to be true. They form part of a script that relies on adages for which we have become accustomed: simulation provides a psychologically safe space to rehearse skills, to make mistakes and to avoid patient harm. But just because these statements *can* be true does not mean that they always are true.

This is by no means the first paper that has challenged some of the conventions, myths and practices that have been enthusiastically adopted in health simulation practice and research, and likely won’t be the last. Further to critiquing the problems or the debate about the problems that exist in simulation practice, this paper seeks to explore the principles and practice of simulation through a different lens. The lens we will look through may generate deeper consideration of why some approaches to working with participants in simulations and simulation programmes work better than others (consider in situ vs non-in situ simulations, variation in approach of debriefers, longitudinal debriefing, perceptions of psychological safety) and how we can improve and optimise our simulation learning environments.

Just as the practice of designing and delivering simulation has been evolving to meet the needs of learners and institutions, so too has the research in this field. As with all other previously emergent fields of research, there is an imperative to (a) reflect on how the quality and direction of research endeavours can be strengthened and (b) act on recommendations that will allow the field to fulfil its potential. In their 2022 editorial, Walter Eppich and Gabriel Reedy note the general change of direction in health simulation research as moving away from aims that seek to justify simulation activities to those which seek to clarify how, and in which circumstances, simulation is effective [2]. Their call to action is clear and framed by three guiding principles:

1. Theoretical frameworks and concepts must be better integrated into all phases of the design and execution of research projects and programmes of research.
2. Varied methodologies and methodological lenses are required to progress the field.
3. Innovative techniques for data collection and analysis should be explored and embraced.

We have been challenged, as a simulation research community, to more deeply consider theory, methodology and methods as they relate to health simulation and health simulation research [2].

The theory that is the focus of this paper is self-determination theory (SDT). SDT focuses on human motivation and behaviours and has informed the growth of various fields [3, 4]. Whilst referred to in some health simulation literature [5–7], it has yet to be comprehensively applied, explored or tested in this field. This paper forms a foundation for discussing some promising lines of research enquiry that could help advance the field of health simulation. It offers an overview of a theoretical framework that appears to be both relevant to health simulation and that offers a variety of methodologies to explore simulation for new insights and areas for practice improvement.

### The theory

Self-determination theory (SDT) is described as a macro theory (in this instance, an overarching theory) of human motivation [1]. First proposed in the 1970s by Richard Ryan and Edward Deci, it has been broadly applied, explored and tested in numerous settings and populations, including primary and secondary schools [8, 9], universities [10–12], workplaces and various health contexts [13].

The origins of SDT lie in the exploration of human motivation and the conditions and environments that impact human behaviours [14]. Over the past four decades, it has slowly and organically developed into a broader theory, which now includes six related “mini-theories” [14]. The mini-theories of SDT include the following: cognitive evaluation theory, organismic integration theory, Causality Orientations Theory, Basic Needs Theory, Goal Content Theory and the Relationships Motivation Theory [15–17] (see Table 1). These interrelated theories offer numerous opportunities for considering foundational principles that may already, and perhaps ought to, underpin the design and delivery of health simulation activities and programmes.

**Table 1:** Overview of the six mini-theories of self-determination theory (SDT)

Theory	Focus	In brief
Cognitive evaluation theory	Intrinsic motivation	Examines supporting and/or undermining factors relating to intrinsic motivation (for example different types and quantities of reward)
Organismic integration theory	Extrinsic motivation	Examines the different qualities of extrinsic motivation
Basic psychological needs theory	Autonomy, competence and relatedness	Examines these three basic psychological needs and how they are associated and involved with motivation, psychological well-being and ill-being
Causality orientation theory	Individual variations	Examines the differences in individual experiences as they relate to social context
Goal contents theory	Intrinsic and extrinsic goals	Examines how the type of life goals people strive for (i.e. intrinsic vs extrinsic) will shape people’s attitudes and behaviours and influence well-being in systematic ways
Relationship motivation theory	Relatedness (basic psychological need)	Examines relational dynamics within the framework of SDT and the further understanding of what motivates and sustains people in relationships

[3, 18]

One of the early propositions in SDT was that the motivations that lead to behaviours (or inaction) could be separated into categories: those that are self-determined (“i.e. governed by the process of choice and experienced as emanating from the self”) and those that are initiated or determined by factors external to the self (“i.e. governed by the process of compliance and experienced as compelled by some interpersonal or intrapsychic force”) [19]. These have come to be known as intrinsic and extrinsic motivational forces.

In SDT, the identified types of motivation are often visualised on a spectrum. At the upper end of this spectrum lies “intrinsic motivation” — behaviours that emanate from a sense of self and that are inherently satisfying [15]. This is followed by four states of “extrinsic motivation”: external regulation, introjection, identification and integration [1]. Finally, at the lower end of the spectrum lies “amotivation” — a state where an individual lacks any intention to act [1, 13].

Intrinsic motivation is explored in the first mini-theory of SDT: *cognitive evaluation theory* — a theory that is concerned with the factors that either undermine or support intrinsic motivation [15]. Intrinsic motivation is described in this theory as a type of self-determined motivation. It is a construct that “describes [a] natural inclination toward assimilation, mastery, spontaneous interest, and exploration” [20]. It has long been acknowledged as developing in humans from birth and, operationally, describes behaviour adopted for its inherently satisfying results [15]. Notably, enjoyment that stems from intrinsic motivation is likely to be conducive to personal growth and eudaimonia — a state of living a “complete” life or “living life well” [21]. Also to note, experiments undertaken in the pursuit of exploring this theory have found that some types of rewards can decrease people’s intrinsic motivation [3].

The aim of all behaviour is most unlikely to be all intrinsically motivated — there are countless internal and external pressures that prompt various behaviours [22]. Whilst intrinsically motivated behaviours are *a* significant type of self-determined behaviours, they are not the only form of self-determined behaviours. There are numerous extrinsically motivated behaviours that are also said to be self-determined. These are further explored in *organismic integration theory*, which posits that distinct characteristics of various extrinsically motivated behaviours can be identified [18].

Extrinsically motivated behaviours are those that are undertaken to obtain an external outcome (for example wealth, notoriety or material goods) [23]. In SDT, the study of extrinsic motivation has been much more concerned with the *quality* of motivation, as opposed to the *quantity* of motivation [23, 24]. This is in contrast to other theories of motivation, particularly as they relate to employment, which often focus on the quantity of motivation that individuals possess in relation to particular tasks [23]. Opportunities to consider this spectrum in health simulation are explored below and culminate in some hypothesised example statements in Table 2.

**Table 2:** Examples of motivational construct statements

	**Construct**	**Description**	**Example statements from literature**	**Possibly relevant statements to provision of health care**	**Possibly relevant statements to health simulation**
**More internalised**	Intrinsic motivation	*Internally rewarding activities that people engage in without needing external prompts or rewards* [3]	“I exercise because it is fun and pleasurable”	[Unknown, but likely not to be intrinsically motivated]	“I participate in simulation activities because they are fun and pleasurable”
	Integrated regulation		“I exercise because I consider exercise a fundamental part of who I am”	“I gain consent from patients because I consider this process to a fundamental part of who I am”	“I participate in simulation activities because I consider participating in this type of activity to be a fundamental part of who I am”
	Identified regulation	*A conscious valuing of a behavioural goal or regulation, such that the action is accepted or owned as personally important* [20]	“I exercise because I value the benefits of exercising”	“I gain consent from patients because I value the benefits of patients being informed prior to making a decision about their care”	“I participate in simulation activities because I value the benefits of participating”
**Less internalised**	Introjected regulation	*Behaviours are performed to avoid guilt or anxiety or to attain ego enhancements such as pride *[20]	“I exercise because I will feel guilty when I don’t”	“I gain consent from patients because I will feel guilty if I don’t”	“I participate in simulation activities because I will feel guilty if I don’t participate”
	External regulation	*Performed to satisfy an external demand or reward contingency* [20]	“I exercise because my physician says I should”	“I gain consent from patients because the legal department says I should”	“I participate in simulation activities because my line manager tells me that I should”
	Amotivation		“I can’t see why I should bother exercising”	“I can’t see why I should bother gaining consent from patients”	“ I can’t see why I should bother participating in simulation activities”

[13]

Ordered from the least to the most internalised of the four subcategories of extrinsic motivation are as follows: *external regulation*, *introjection*, *identification* and *integration* (see descriptions in Table 2). These lie on a continuum of self-determination and, when exercised, produce demonstrably different outcomes and associated outputs. External regulation and introjected motivation are forms of “non-self-determined” motivation [15, 23]. Behaviours that fall into the category of extrinsic motivation are regulated by an external pressure or an external reward, such as financial remuneration or threat of punishment [13, 25]. Identification and integration are considered to be autonomous and self-regulated forms of extrinsic motivation [15, 25].

Identifying that there are qualitative differences that underlie peoples’ extrinsically motivated behaviours is important [23] (consider your experiences of working with simulation participants who love simulation, versus those who attend because it is a requirement of their job or education). Evaluating these differences holds value for understanding human behaviour, and how our social environment and work systems can be designed to optimise human potential [1].

Amotivation sits at the opposite end of the spectrum from intrinsic motivation. When amotivation is experienced in a workplace, for example, an employee may value an activity or behaviour so little that no effort is exerted to complete or realise the potential of that behaviour [13] (for a health-related example, consider the issues of poor adherence to appropriate hand hygiene).

To illustrate the different constructs of motivation, example statements relating to the qualities of motivation to participating in physical exercise, as presented by Ng et al. [13], are provided in Table 2. Alongside, these sit some potential statements relating to health professionals, and the quality of motivation to gain consent from patients, and examples relating to participating in simulation activities.

SDT is concerned not only with the quality of motivation but also the types of environments and contexts that effect motivation and the changes in motivation people may experience. *organismic integration theory* asserts that people are *inherently* driven towards learning, mastery and connection [16]. This inherent quality, however, is not achieved without conditions that are supportive. These conditions are believed to include three fundamental psychological needs: autonomy, competence and relatedness [24]. Indeed, the presence or absence of conditions that support these basic needs may “sustain [or] diminish the “innate propensity” of humans to act from an intrinsic motivation” [20]. The examination of intrinsic motivation has therefore been one that has evaluated these conditions and forms the basis for *basic psychological needs theory*.

Three basic psychological needs are explored in basic psychological needs theory: autonomy, competence and relatedness. In the context of SDT, autonomy refers to the “the perception of being the origin of one’s own behavior and experiencing volition in action” [13], and is not defined by autonomy’s other definitions which relate to independence and separation [17].

*Autonomy* has been explored at length in terms of both the individual experience and the contexts that either support or inhibit this psychological need [16]. *Autonomy supportive environments* include those that encourage and allow individuals to experience their behaviour as volitional. Features of autonomy supportive environments include nonjudgemental attitudes, the provision of rationales for suggestions or decisions and the facilitation of self-regulation [26].

*Competence* is described as “the feeling of being effective in producing desired outcomes and exercising one’s capacities” [13]. It is concerned with mastery [16]. It has been identified that “the need for competence is best satisfied within well-structured environments that afford optimal challenges, positive feedback and opportunities for growth” [16]. It is not hard to draw links between this statement and the practice of health simulation. We can hypothesise that the high levels of satisfaction students report when participating in simulation event(s) are inextricably linked to the efforts made to create a structured environment and to provide feedback through debriefing that is both positive and directive for growth.

*Relatedness* is defined in SDT as the “feeling of being respected, understood, and cared for by others” [13]. *Relationship motivation theory* is the newest of the six mini-theories and focuses on the impact of basic psychological needs on interpersonal relationships. A central idea in Relationship Motivational Theory is *mutuality of autonomy* [3]. In other words, the equal creation of autonomy-supportive environments from each party. This idea has interesting implications for the relationship that develops between facilitators and participants and indeed between participants themselves.

### How has SDT already been applied to simulation?

A handful of studies have been published that investigate links between elements of SDT and the design of health simulation scenarios, activities and programmes. Table 3 provides a brief overview of the studies which have identified SDT itself or elements of SDT in their study. They include three prospective, quantitative studies [25, 27, 28]; two mixed-methods studies [29, 30] and one qualitative study [5]. SDT was also mentioned in a discussion paper regarding mastery learning, but not extensively explored [6].

**Table 3:** Examples of SDT in current simulation and medical education literature

**Authors (year of publication)**	**Study aim (verbatim)**	**Methods**	**Participants**	**Brief overview of findings**
Diaz-Agea, Pujalte-Jesus [5]	The aim of this study was to explore the views and perspectives of students involved in simulation-based learning related to their process of motivation. Also, to identify the motivational elements they perceived, as well as the aspects that could reduce their motivation in the simulation sessions	Qualitative study Focus-group discussions	101 nursing students	Various themes and subthemes were identified and groups into factors that participants identified as motivating and demotivating and suggest that these could be leveraged to motivate students to participate in their nursing education to a greater extent
Escher, Rystedt [30]	The aims of this study were twofold: First, to examine the responses of the professional groups involved in the training, particularly issues related to the development of self-efficacy and situational motivation. Second, [the authors] wanted to explore participants’ perceptions of the design features important to the training and the opportunities for and barriers to transferring the lessons learned in [simulation-based team training] to teamwork in the operating room	Mixed methods Self-efficacy questionnaire SIMS survey Focus-group discussions	71 health professionals who work in operating theatres	The team training provided was associated with increased self-reported confidence and intrinsic motivation in the operating room team members who participated. Barriers to transferring lessons learned into clinical practice largely related to organisational/system level factors
Henry, Vesel [29]	The overall goal of [the] study was to gain information on how educational environments can promote feedback seeing among learners	Mixed methods IMI survey + participant interviews	34 medical residents completed IMI survey 10 interviews	The relationship between motivation and feedback is complex. The IMI could not predict this relationship, and factors other than motivation were linked to feedback-seeking behaviours
Moll-Khosrawi, Cronje [25]	Hypothesis testing: [Authors] hypothesised that [simulation-based medical education] and bedside teaching enhance autonomous motivation and decrease controlled motivation	Prospective interventional cohort study design SIMS	145 third-year medical students sampled, with varied response rate at different time points	In participants who had bedside teaching, there was found to be a decrease in external (controlled) motivation and identified (autonomous) motivation. The simulation-based trainings did not change students’ level of motivation
Schulte-Uentrop, Cronje [27]	[Authors] investigated the correlation of students’ motivation and their performance of non-technical skills during simulation-based emergency training (SBET)	Prospective cross-sectional cohort study SIMS Anaesthesiology students’ non-technical skills	422 medical students (years 1–4)	Higher levels of autonomous situational motivation did not correlate with better performance in non-technical skills during the SBET
Thoma, Hayden [28]	The purpose of this study was to determine whether first-year medical students reported greater intrinsic motivation when participating in higher autonomy simulation sessions as compared with lower autonomy sessions	Non-randomised crossover trial Adapted IMI survey	22 first-year medical students	Extracurricular sessions increased participants’ perceived autonomy, but they were highly intrinsically motivated in both settings

Abbreviations: *IMI* inventory of intrinsic motivation, *SBET* simulation-based emergency training, *SIMS* situational motivation

As can be seen in Table 3, the aims and hypotheses being explored are somewhat varied, but all have a focus on motivation. For example, in the studies conducted by Diaz-Agea, Pujalte-Jesus [5] and Escher and Rystedt [30], motivation to participate in the simulations themselves is explored in cohorts of nursing students and health professionals respectively. In the Henry and Vesel [29] example, motivation was explored in relation to participants’ feedback-seeking behaviours. Autonomy is the other SDT element that is explored, with studies working to determine its relationship with different types of motivation [25, 28].

Two questionnaires that have been developed in the exploration of SDT were used in the studies included in Table 3: The inventory of intrinsic motivation (IMI) scale and the Situational Motivation Scale (SIMS). The IMI derives from one of the mini-theories of SDT: cognitive evaluation theory [31]. There are numerous versions of this questionnaire which have been adapted for different contexts (e.g. sport, physical education) and experiments which have tested cognitive evaluation theory [28, 31]. The SIMS is a validated tool that invites participants to respond to prompts linked to four types of motivation: intrinsic motivation, identified regulation, external regulation and amotivation [32].

Beyond the discrete cases of SDT being investigated in health simulation in the examples provided, there is no current programme of research that is exploring this theory in relation to health simulation. A broader and deeper exploration of the theory and its relevance to health simulation is warranted. It is warranted because of the following: (1) there is a necessity for our field to better understand theoretical foundations that may facilitate progress, and appropriate reform, in the design and delivery of simulation, (2) there is a growing demand for theory to underpin our own professional development as simulationists [2, 33], (3) there is potential for this deeper understanding of practice to enhance outcomes for learners and patients and (4) there are existing parallels between the language used in the study of SDT and the practice of health simulation.

### Current and future implications

We have opportunities to more deeply consider the fundamental principles that underpin health simulation and to determine what elements of SDT could lead to improvements in the design and delivery of health simulation activities, programmes and research.

The conceptual argument for this is founded in some assumptions. Namely, that SDT (1) is a relevant theory to consider when exploring how and why simulation is an effective modality for technical and behavioural skill development in the health simulation context, (2) offers new avenues for exploring how simulations can be designed with enhanced and predictable participant benefit, (3) may be relevant in explaining why people who deliver simulation activities (including simulated patients, embedded participants and simulation coordinators) value participating in this type of activity and (4) has the potential to explain why simulation is a successful modality for learning, skill development, team building and for improving system functionality and safety.

At face value, it does appear that the principles that have guided health simulation activities can be firmly linked to foundational components of SDT. If we consider the often adopted “basic assumption”, through the lens of SDT, we can see alignment between language and theory: (“We believe that everyone participating in this simulation is intelligent, capable [competence], cares about doing their best [autonomy, competence, motivation] and wants to improve [motivation]”) [34].

In moving from an intuited to an explicit practice of psychological safety that is founded in SDT, we can apply evidence from the broader health professions and clinical education literature. This literature strongly suggests psychological safety can be provided and optimised when an “autonomy supportive” environment is created and sustained [11, 35]. The benefits of autonomy supportive environments include the increased intrinsic motivation of learners (i.e. learners experience deep satisfaction in the learning process and are intrinsically motivated to continue that learning process). Examples of how the features of autonomy supportive environments may already, or could, be applied to health simulation are outlined in Table 4.

**Table 4:** Example features of autonomy-supportive environments, as applied to health simulation

Domain	Feature	Application to health simulation
**Autonomy**	Provide meaningful rationales for learning activities [11, 36]	Learning outcomes are explicit and linked to a curriculum
	Minimise pressure and control [11, 36]	Learners have choice in participating in scenarios
	Encourage active participation [37]	Learners are encouraged to participate in scenarios in different roles and to actively engage in debrief discussions
	Acknowledge feelings of learners [11, 36]	Debrief facilitates acknowledgement of emotions experienced in scenario
	Encourage learners to accept responsibility for their learning [11, 12]	Learners are held accountable for their role and for the outcomes of the simulation
**Competence**	Provide optimally challenging tasks [11, 37]	Learners are challenged to work at their zone of proximal development
	Provide structured guidance [11]	Learners are aware of the structure of discrete simulations (e.g. pre-brief, scenario, debrief), the simulation programme in which they are participating, and the intended goals of the simulation (e.g. personal, team or translational/system goals)
	Entrust learners with more clinical responsibilities [11]	Learners are entrusted with sufficiently challenging responsibilities throughout the simulation program
	Provide constructive feedback [11]	Constructive feedback is provided in the debrief
**Relatedness**	Convey warmth and respect to learners [11, 12]	Simulation facilitators are collegial and respectful of learners at all stages of simulation-based education and of all participants from all professions, disciplines and teams
	Provide acknowledgement and support for expressions of negative affect/emotion [11, 37]	Students are supported to understand, manage and express emotion throughout their simulation experiences
	Provide nonthreatening teaching environment [11]	Information is provided about the intent, benefits and expectations of the simulation environment, so that perceptions of threat to reputation or safety are minimised
	Provide feedback and opportunities for reflective practice [11]	Learning opportunities are provided for the purpose feedback and reflective practice throughout simulation events and programmes — this would be particularly relevant in the debrief

In efforts to understand the foundations of good quality health simulation, and to further explore the validity of the various components of SDT in this field, research projects can address quite a broad array of questions. It would be relevant to examine how SDT could further inform simulation design and delivery (as described above), simulation participants’ *quality* of motivation to transfer technical and behavioural skills to the clinical environment, how principles and evidence from SDT could be incorporated into faculty development and how performance can be optimised.

As an example, we can consider practitioners’ *quality of motivation* to gain patients’ consent. Gaining informed consent is a fundamental part of working as a health professional [38]. We know that patients are not optimally providing informed consent for procedures [39], nor for participating in medical research (e.g. pharmaceutical trials) [40]. There are acknowledged issues related to patients’ level of health literacy and clinicians’ overconfidence that patients have understood what they have explained, and there is an opportunity to examine the role of education and performance enhancement in addressing these issues [39]. SDT could be used to examine health professionals’ quality of motivation for gaining informed consent. Relevant, preliminary research questions include the following: “What is the quality of motivation that health professions students and health professionals demonstrate in relation to the technical and behavioural skills of gaining informed consent from patients” and “What influences health professionals’ quality of motivation for gaining consent?”.

When considering how to apply this knowledge into the design of a simulation, we can ask questions about the impact of different approaches for learning about the consent process. “Is externally regulated motivation to gain informed consent related to learning about this process from a predominantly legal perspective?” “Does learning/reflecting on these skills from a bioethics perspective lead to identified or integrated motivation when gaining consent in a simulated scenario?” “What are the intended and un-intended consequences for participants who have come to simulations from these different teaching perspectives?” Given previous work with SDT, we might hypothesise that learners will be impacted by these external factors, and their subsequent behaviours may be moderated by the lens of teaching or debriefing that is adopted. This same principle would apply to an array of technical and behavioural skills — hand hygiene, breaking bad news, engaging in low dose and high-frequency simulation for the maintenance of various skills.

Pathways exist for investigating the relevance of SDT to health simulation and for testing SDT theory in simulated contexts. These can be shaped to further extend the work of others who have investigated SDT and to provide evidence to underpin the various techniques and modalities of health simulation.

Ultimately, we should be aiming to generate and then to use the best available evidence to support simulation practice, support the refinement of learning outcomes and support faculty development efforts. SDT is a theory that has been built and tested slowly, strategically and with care not to oversimplify concepts or to foster reductionism. What we can work towards is not just isolated studies that may lead to another set of education myths [41, 42]. We have the opportunity to continue in the SDT tradition of systematically testing ideas and theory to determine if and what principles will facilitate a maturing of health simulation for teaching, training, systems testing, performance evaluation and professional development.

## Conclusion

SDT is a theory that has been explored in many fields, and whilst elements have been explored in simulation, this exploration is in its infancy. Proposed in this paper is a rationale for conducting research that examines the relevance of the theory to health simulation and explore how health simulation may benefit from SDT research from other fields.

Why might we do this? We come back to the introduction of this paper where we consider the statements and philosophy that we want to be true in the field of health simulation. There is a pathway for testing our underlying assumptions and to enhancing our practice through detailed, structured and theoretically sound methods. In testing potential associations between SDT and simulation, we may be better informed about when statements we make are more likely to be true (“this is a psychologically safe environment”) and when they really may not be. In examining health simulation through the lens of SDT, we have opportunities to capture new insights into *why* simulation can be effective in enhancing performance and to further generate an evidence base for best practice in this field.

## Data Availability

Not applicable.

## Abbreviations

IMI: Inventory of intrinsic motivation
SDT: Self-determination theory
SBET: Simulation-based emergency training
SIMS: Situational Motivation Scale

## References

[1] Ryan RM, Deci EL, Vansteenkiste M, Soenens B. Building a science of motivated persons: self-determination theory’s empirical approach to human experience and the regulation of behavior. Motivation Science. 2021;7(2):97–110. 10.1037/mot0000194

[2] Eppich W, Reedy G. Advancing healthcare simulation research: innovations in theory, methodology, and method. Adv Simul (Lond). 2022;7(1):23. 35897062 10.1186/s41077-022-00219-y PMC9326413

[3] Ryan RM, Deci EL. Brick by brick: the origins, development, and future of self-determination theory. Advances in Motivation Science. 2019. p. 111–56.

[4] Deci EL, Ryan RM. Intrinsic motivation and self-determination in human behavior. New York: Springer Science+Business Media New York; 1985.

[5] Diaz-Agea JL, Pujalte-Jesus MJ, Leal-Costa C, Garcia-Mendez JA, Adanez-Martinez MG, Jimenez-Rodriguez D. Motivation: bringing up the rear in nursing education. Motivational elements in simulation. The participants’ perspective. Nurse Educucation Today. 2021;103:104925. 10.1016/j.nedt.2021.104925 33962187

[6] Eppich WJ, Hunt EA, Duval-Arnould JM, Siddall VJ, Cheng A. Structuring feedback and debriefing to achieve mastery learning goals. Acad Med. 2015;90(11):1501–8. 26375272 10.1097/ACM.0000000000000934

[7] Sørensen J, Van der Vleuten C, Lendschou J, Gluud C, Østergaard D, LeBlanc V, et al. ‘In situ simulation’ versus ‘off site simulation’ in obstetric emergencies and their effect on knowledge, safety attitudes, team performance, stress, and motivation: study protocol for a randomized controlled trial. Trials. 2013;14(220):1–11. 23870501 10.1186/1745-6215-14-220 PMC3716971

[8] Taylor G, Jungert T, Mageau GA, Schattke K, Dedic H, Rosenfield S, et al. A self-determination theory approach to predicting school achievement over time: the unique role of intrinsic motivation. Contemp Educ Psychol. 2014;39(4):342–58. 10.1016/j.cedpsych.2014.08.002

[9] Froiland JM, Worrell FC. Intrinsic motivation, learning goals, engagement, and achievement in a diverse high school. Psychol Sch. 2016;53(3):321–36. 10.1002/pits.21901

[10] Orsini C, Binnie VI, Wilson SL. Determinants and outcomes of motivation in health professions education: a systematic review based on self-determination theory. Journal of Education Evaluation for Health Professionals. 2016;13:19. 10.3352/jeehp.2016.13.19 PMC4863137 27134006

[11] Orsini C, Evans P, Jerez O. How to encourage intrinsic motivation in the clinical teaching environment?: a systematic review from the self-determination theory. Journal of Education Evaluation for Health Professionals. 2015;12:8. 10.3352/jeehp.2015.12.8 PMC4397857 25855386

[12] Ten Cate TJ, Kusurkar RA, Williams GC. How self-determination theory can assist our understanding of the teaching and learning processes in medical education. AMEE guide No. 59. Med Teach. 2011;33(12):961–73. 22225433 10.3109/0142159X.2011.595435

[13] Ng JY, Ntoumanis N, Thogersen-Ntoumani C, Deci EL, Ryan RM, Duda JL, et al. Self-Determination Theory applied to health contexts: a meta-analysis. Perspect Psychol Sci. 2012;7(4):325–40. 26168470 10.1177/1745691612447309

[14] Ryan RM, Soenens B, Vansteenkiste M. Reflections on self-determination theory as an organizing framework for personality psychology: Interfaces, integrations, issues, and unfinished business. J Pers. 2019;87(1):115–45. 30325499 10.1111/jopy.12440

[15] Vansteenkiste M, Niemiec CP, Soenens B. The development of the five mini-theories of self-determination theory: an historical overview, emerging trends, and future directions. The Decade Ahead: Theoretical Perspectives on Motivation and Achievement. Advances in Motivation and Achievement. 2010. p. 105–65.

[16] Ryan R, Deci E. Intrinsic and extrinsic motivation from a self-determination theory perspective: definitions, theory, practices, and future directions. Contemporary Educational Psychology. 2020;61.

[17] Sheldon KM, Prentice M. Self-determination theory as a foundation for personality researchers. J Pers. 2019;87(1):5–14. 29144550 10.1111/jopy.12360

[18] Reeve J, Lee W. A neuroscientific perspective on basic psychological needs. J Pers. 2019;87(1):102–14. 29626342 10.1111/jopy.12390

[19] Deci EL, Eghrari H, Patrick BC, Leone DR. Facilitating internalization: the self-determination theory perspective. J Pers. 1994;62(1):119–42. 8169757 10.1111/j.1467-6494.1994.tb00797.x

[20] Ryan R, Deci E. Self-determination theory and the facilitator of intrinsic motivation, social development, and well-being. Am Psychol. 2000;55(1):68–78. 11392867 10.1037/0003-066X.55.1.68

[21] Ryan RM, Huta V, Deci EL. Living well: a self-determination theory perspective on eudaimonia. J Happiness Stud. 2006;9(1):139–70. 10.1007/s10902-006-9023-4

[22] Ryan RM, Koestner R, Deci EL. Ego-involved persistence: when free-choice behaviour is not intrinsically motivated. Motiv Emot. 1991;15(3):185–205. 10.1007/BF00995170

[23] Van den Broeck A, Howard JL, Van Vaerenbergh Y, Leroy H, Gagné M. Beyond intrinsic and extrinsic motivation: a meta-analysis on self-determination theory’s multidimensional conceptualization of work motivation. Organ Psychol Rev. 2021;11(3):240–73.

[24] Teixeira PJ, Marques MM, Silva MN, Brunet J, Duda JL, Haerens L, et al. A classification of motivation and behavior change techniques used in self-determination theory-based interventions in health contexts. Motivation Science. 2020;6(4):438–55. 10.1037/mot0000172

[25] Moll-Khosrawi P, Cronje JS, Zollner C, Kubitz JC, Schulte-Uentrop L. Understanding how the motivational dimension of learning is influenced by clinical teaching in medical education: a prospective cohort study. Annals of Medicine and Surgery. 2021;65: 102366. 34007448 10.1016/j.amsu.2021.102366 PMC8111262

[26] Ryan RM, Deci EL. A self-determination theory apporach to psychotherapy: the motivational basis for effective change. Canadian Psychology / Psychologie canadienne. 2008;49(3):186–93. 10.1037/a0012753

[27] Schulte-Uentrop L, Cronje JS, Zollner C, Kubitz JC, Sehner S, Moll-Khosrawi P. Correlation of medical students’ situational motivation and performance of non-technical skills during simulation-based emergency training. BMC Med Educ. 2020;20(1):351. 33032572 10.1186/s12909-020-02247-6 PMC7542687

[28] Thoma B, Hayden EM, Wong N, Sanders JL, Malin G, Gordon JA. Intrinsic motivation of preclinical medical students participating in high-fidelity mannequin simulation. BMJ Simulation Technology and Enhanced Learning. 2015;1(1):19–23. 10.1136/bmjstel-2015-000019 PMC8936958 35517845

[29] Henry D, Vesel T, Boscardin C, van Schaik S. Motivation for feedback-seeking among pediatric residents: a mixed methods study. BMC Med Educ. 2018;18(1):145. 29921262 10.1186/s12909-018-1253-8 PMC6007008

[30] Escher C, Rystedt H, Creutzfeldt J, Meurling L, Hedman L, Fellander-Tsai L, et al. All professions can benefit - a mixed-methods study on simulation-based teamwork training for operating room teams. Adv Simul. 2023;8(1):18. 10.1186/s41077-023-00257-0 PMC10351117 37460943

[31] Ryan RM, Mims V, Koestner R. Relation of reward contingency and interpersonal context to intrinsic motivation: a review and test using cognitive evaluation theory. J Pers Soc Psychol. 1983;45(4):736–50. 10.1037/0022-3514.45.4.736

[32] Guay F, Vallerand RJ, Blanchard C. On the assessment of situational intrinsic and extrinsic motivation: the Situational Motivation Scale (SIMS). Motiv Emot. 2000;24(3):175–213. 10.1023/A:1005614228250

[33] Gardner AK, Rodgers DL, Steinert Y, Davis R, Condron C, Peterson DT, et al. Mapping the terrain of faculty development for simulation: a scoping review. Simul Healthc. 2024;19(1S):S75–89. 38240621 10.1097/SIH.0000000000000758

[34] Sawyer T, Eppich W, Brett-Fleegler M, Grant V, Cheng A. More than one way to debrief: a critical review of healthcare simulation debriefing methods. Simulation in Healthcare. 2016;11(3):209–17. 27254527 10.1097/SIH.0000000000000148

[35] Reeve J, Cheon SH. Autonomy-supportive teaching: its malleability, benefits, and potential to improve educational practice. Educational Psychologist. 2021;56(1):54–77. 10.1080/00461520.2020.1862657

[36] Niemiec CP, Ryan RM. Autonomy, competence, and relatedness in the classroom. Theory Res Educ. 2009;7(2):133–44. 10.1177/1477878509104318

[37] Kusurkar RA, Croiset G, Ten Cate TJ. Twelve tips to stimulate intrinsic motivation in students through autonomy-supportive classroom teaching derived from self-determination theory. Med Teach. 2011;33(12):978–82. 22225435 10.3109/0142159X.2011.599896

[38] Cetina-Sauri G, Huchim-Lara O, Alvarez-Baeza A, Inurreta-Díaz M, Puga-Matu H, Aguilar-Vargas E, et al. Undergraduate medical students. Simulation-based activity to conduct the informed consent process for health research studies. Educación Médica. 2020;21(2):106–11. 10.1016/j.edumed.2018.05.015

[39] Ochieng J, Buwembo W, Munabi I, Ibingira C, Kiryowa H, Nzarubara G, et al. Informed consent in clinical practice: patients’ experiences and perspectives following surgery. BMC Res Notes. 2015;8:765. 26653100 10.1186/s13104-015-1754-z PMC4675036

[40] Pietrzykowski T, Smilowska K. The reality of informed consent: empirical studies on patient comprehension-systematic review. Trials. 2021;22(1):57. 33446265 10.1186/s13063-020-04969-w PMC7807905

[41] Molloy E, Ajjawi R, Bearman M, Noble C, Rudland J, Ryan A. Challenging feedback myths: values, learner involvement and promoting effects beyond the immediate task. Med Educ. 2020;54(1):33–9. 31475387 10.1111/medu.13802

[42] Norman G. The once and future myths of medical education. J Grad Med Educ. 2020;12(2):125–30. 32322339 10.4300/JGME-D-20-00185.1 PMC7161332

